# Supplementary material for: CK2-mediated phosphorylation of SUZ12 promotes PRC2 function by stabilizing enzyme active site
Source: Nat Commun. 2022 Nov 9;13:6781. doi: 10.1038/s41467-022-34431-1 (PMC9645763; doi:10.1038/s41467-022-34431-1)
Supplement: Supplementary file 1 — Supplementary Information [file 41467_2022_34431_MOESM1_ESM.pdf]

Supplementary Fig. 1

Gong L et al.

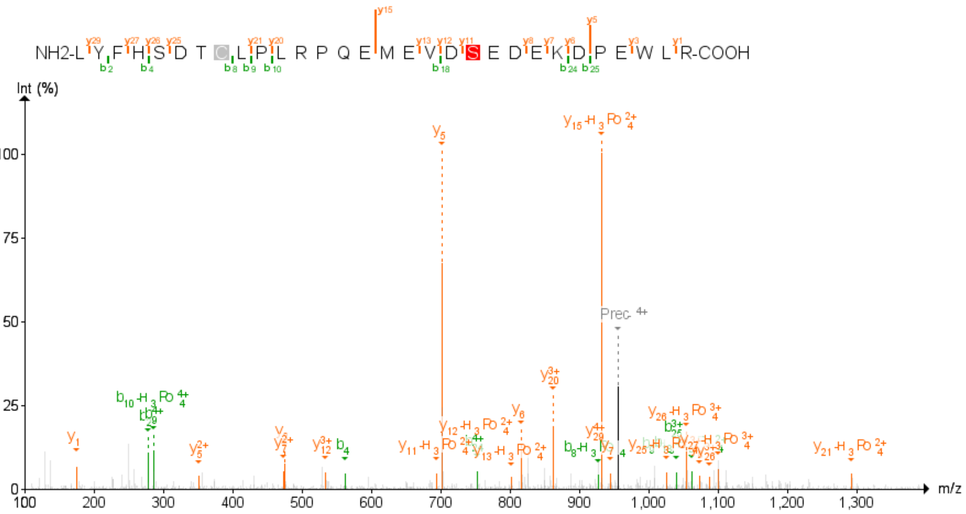

Supplementary Fig. 1. MS/MS spectrum showing phosphorylation on mSUZ12 S585-containing peptide

The peptide sequence is LYFHSDTCLPLRPQEMEVD**S**EDEKDPEWLR. Y ions are shown in orange and b ions in green.

Supplementary Fig. 2

Gong L et al.

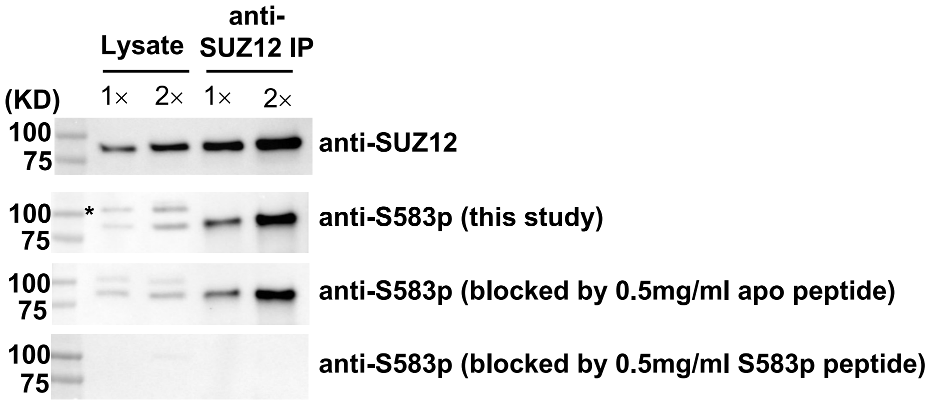

Supplementary Fig. 2. Specificity of anti-SUZ12S583p antibody

Antibody specificity is confirmed by peptide blocking of Western signals. Asterisk indicates a nonspecific protein with an apparent molecular weight higher than SUZ12 that was also recognized by the newly developed anti-SUZ12S583p antibody. Immunoprecipitates of anti-SUZ12 antibody did not contain this protein and were used to generate clean anti-SUZ12S583p signals in this study. Uncropped gel images of the supplemental figures are shown in Figure S19. A representative of two independent experiments is shown.

Source data are provided as a Source Data file.

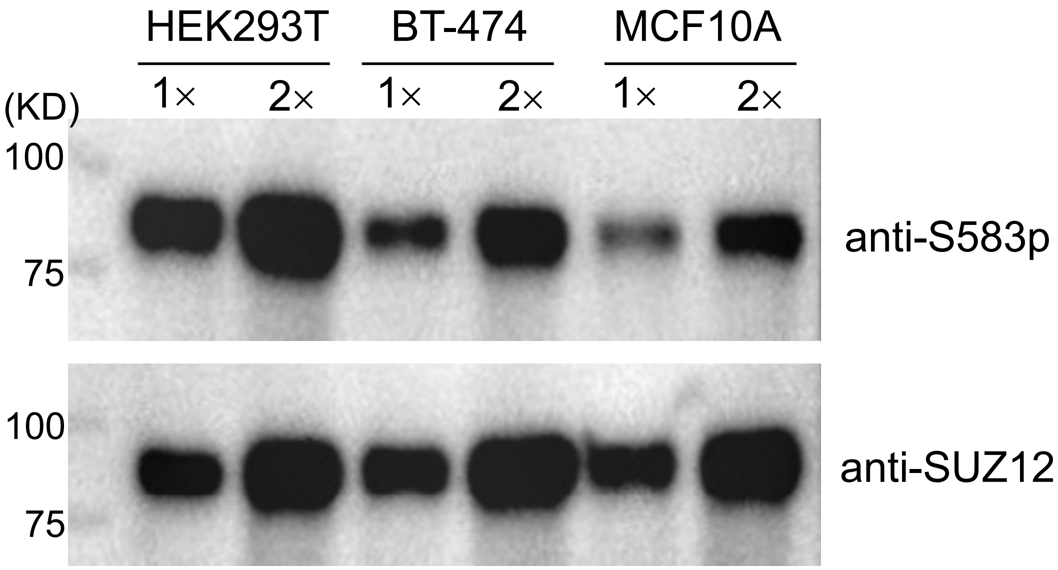
HEK293T
BT-474
MCF10A
1×
2×
1×
2×
1×
2×
(KD)
100
75
anti-S583p
100
75
anti-SUZ12

Supplementary Fig. 3. Differential phosphorylation of SUZ12S583 in cell lines

Levels of S583 phosphorylation in HEK293T cells, BT-474 breast cancer cells, and MCF10A breast epithelial cells. Anti-SUZ12S583p signals were generated using immunoprecipitates of anti-SUZ12 antibody. A representative of two independent experiments is shown.

Source data are provided as a Source Data file.

Supplementary Fig. 4 Gong L et al.

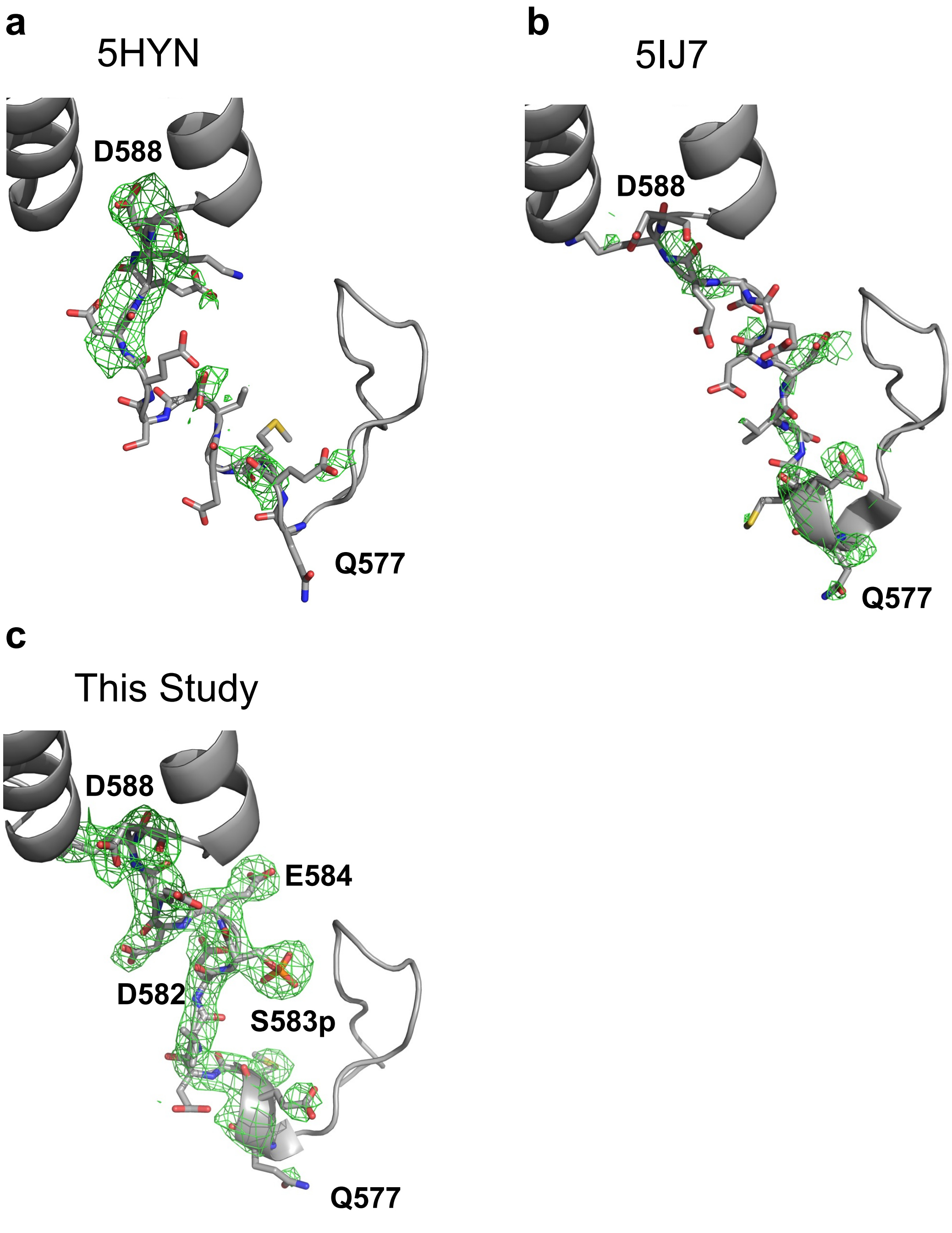

Supplementary Fig. S4. F_oF_c omit map of PRC2 structures

The PDS loop (residues 577–588) of SUZ12 was omitted from the current structure and two other PRC2 structures with comparable resolutions at 2.6–3.0 Å (5HYN and 5IJ7). F_oF_c omit map was generated in autoBUSTER. Images were rendered in PyMOL and electron densities shown were contoured at 2.5 σ.

Supplementary Fig. S5

Gong L et al.

Peptide Sequence	Modifications	PSMs	Abundance
[R].LYFHSDTCLPLRPQEMEV DSEDEKDPEWLR.[E]	1xCarbamidomethyl [C8]; **1xPhospho [S20]**	71	13567667690
[R].LYFHSDTCLPLRPQEMEV DSEDEKDPEWLREK.[T]	1xCarbamidomethyl [C8]; **1xPhospho [S20]**; 1xOxidation [M16]	57	4145004012
[R].LYFHSDTCLPLRPQEMEV DSEDEKDPEWLR.[E]	1xCarbamidomethyl [C8]; **2xPhospho [S20; T/S]**; 1xOxidation [M16]	2	8479983
[R].LYFHSDTCLPLRPQEMEV DSEDEKDPEWLREK.[T]	1xCarbamidomethyl [C8]; **2xPhospho [S5; S20]**; 1xOxidation [M16]	1	0 (below limit of quantitation)
[R].LYFHSDTCLPLRPQEMEV DSEDEKDPEWLR.[E]	1xCarbamidomethyl [C8]; **1xPhospho [S/T]**; 1xOxidation [M16]	63	5328943898
[R].LYFHSDTCLPLRPQEMEV DSEDEKDPEWLREK.[T]	1xCarbamidomethyl [C8]; **1xPhospho [T/S/Y]**	57	5691230830
[R].LYFHSDTCLPLRPQEMEV DSEDEKDPEWLR.[E]	1xCarbamidomethyl [C8]; 1xOxidation [M16]	5	147456498
[R].LYFHSDTCLPLRPQEMEV DSEDEKDPEWLR.[E]	1xCarbamidomethyl [C8]	5	605541175
[R].LYFHSDTCLPLRPQEMEV DSEDEKDPEWLREK.[T]	1xCarbamidomethyl [C8]; 1xOxidation [M16]	4	9193775
[R].LYFHSDTCLPLRPQEMEV DSEDEKDPEWLREK.[T]	1xCarbamidomethyl [C8]	2	797614192

Supplementary Fig. 5. Semi-quantitative analysis of S583 phosphorylation by LC-MS/MS

Peptide sequence, modification, number of peptide spectrum matches (PSMs), and peptide abundance are listed. Phosphorylated residues that were unambiguously assigned are shown in orange. Ambiguous phosphorylation sites are colored in blue. Percentage of phosphorylation could be estimated to be around 58.5% – 94.9% based on a comparison of S583 phosphopeptide abundances to those for unphosphorylated peptides containing S583, assuming that the ionization efficiencies of the phosphorylated and unphosphorylated peptide are the same.

Supplementary Fig. 6

Gong L et al.

a

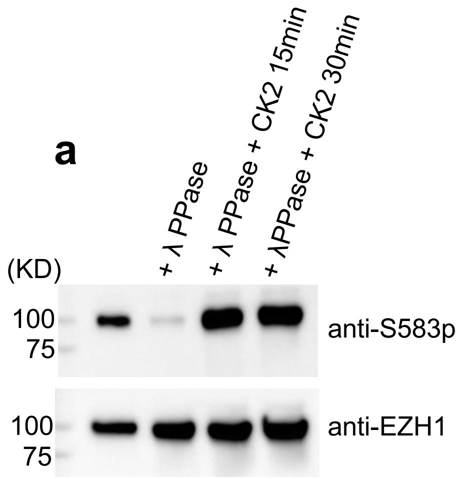

b

Peptide Sequence	Modifications	# PSMs	Protein	Re-phosphorylated	Dephosphorylated
[K].MEDDIDKIKPSESNVTILGR.[F]		21	EED	1.37E+08	1.17E+08
[K].MEDDIDKIKPSESNVTILGR.[F]	1xOxidation [M1]	35	EED	7.56E+07	4.02E+07
[K].MEDDIDKIKPSESNVTILGR.[F]	1xPhospho [S11(90.2)]	2	EED	1.75E+05	1.10E+05
[R].DSSILIAVCDDASIWR.[W]	1xCarbamidomethyl [C9]	72	EED	1.18E+08	6.77E+07
[R].DSSILIAVCDDASIWR.[W]	1xCarbamidomethyl [C9]; 1xPhospho [S2(86.8)]	1	EED	1.70E+04	0 (below limit of quantification)
[R].LYFHSDTCLPLRPQEMEVDSEDEKDPEWLREK.[T]	1xCarbamidomethyl [C8]; 1xOxidation [M16]; 1xPhospho [S20(100)]	15	SUZ12	2.61E+06	2.78E+05
[R].LYFHSDTCLPLRPQEMEVDSEDEKDPEWLR.[E]	1xCarbamidomethyl [C8]	17	SUZ12	1.80E+06	1.68E+07
[R].LYFHSDTCLPLRPQEMEVDSEDEKDPEWLR.[E]	1xCarbamidomethyl [C8]; 1xOxidation [M16]	16	SUZ12	2.18E+06	1.71E+07
[R].LYFHSDTCLPLRPQEMEVDSEDEKDPEWLR.[E]	1xCarbamidomethyl [C8]; 1xPhospho [S20(100)]	45	SUZ12	5.79E+07	8.63E+05
[R].LYFHSDTCLPLRPQEMEVDSEDEKDPEWLR.[E]	1xCarbamidomethyl [C8]; 1xOxidation [M16]; 1xPhospho [S20(100)]	31	SUZ12	2.95E+07	7.38E+05
[R].LYFHSDTCLPLRPQEMEVDSEDEKDPEWLREK.[T]	1xCarbamidomethyl [C8]	5	SUZ12	5.32E+04	3.17E+06
[R].LYFHSDTCLPLRPQEMEVDSEDEKDPEWLREK.[T]	1xCarbamidomethyl [C8]; 1xOxidation [M16]	6	SUZ12	0 (below limit of quantification)	2.37E+06
[R].LYFHSDTCLPLRPQEMEVDSEDEKDPEWLREK.[T]	1xCarbamidomethyl [C8]; 1xPhospho [S20(100)]	8	SUZ12	5.71E+06	7.11E+05

Supplementary Fig. 6. *In vitro* dephosphorylation and re-phosphorylation of the PRC2-EZH1 minimal complex

a. Western blot of the dephosphorylated and re-phosphorylated minimal complex. The PRC2-EZH1 minimal complex expressed in yeast was subjected to *in vitro* dephosphorylation by ***λ*** phosphatase followed by *in vitro* re-phosphorylation by human CK2. ***λ*** phosphatase was removed by size exclusion chromatography prior to re-phosphorylation. A representative of three independent experiments is shown.

Source data are provided as a Source Data file.

b. LC-MS/MS analysis of the dephosphorylated and re-phosphorylated PRC2-EZH1 minimal complex. Phosphorylated serine residues are highlighted in orange. Assuming that the ionization efficiencies of the phosphorylated and unphosphorylated peptide are the same, percentage of phosphorylation could be calculated based on a comparison of the sum of the abundances of the phosphorylated peptides to those of unphosphorylated peptides. Whereas the SUZ12S583 was 6.2% and 96.0% phosphorylated in the dephosphorylated and re-phosphorylated complex, respectively, the phosphorylation level of the two serine residues from EED was less than 0.1%. Over 40 distinct serine residues from the minimal complex were captured by LC-MS/MS, and those without phosphorylation detected are not listed in the table.

Supplementary Fig. S7

Gong L et al.

```
                                      684
EZH1_HUMAN      LKKHLLLAPSDVAGWGTFIKESVQKNEFISEYCGELISQDEADRRGKVYDKYMSSFLFNL 670
EZH2_HUMAN      SKKHLLLAPSDVAGWGIFIKDPVQKNEFISEYCGEIISQDEADRRGKVYDKYMCSFLFNL 669
                 *************** ***: *************:*****************.******

                              684
EZH1_HUMAN      NNDFVVDATRKGNKIRFANHSVNPNCYAKVVMVNGDHRIGIFAKRAIQAGEELFFDYRYS 730
EZH2_HUMAN      NNDFVVDATRKGNKIRFANHSVNPNCYAKVMMVNGDHRIGIFAKRAIQTGEELFFDYRYS 729
                ******************************:*****************:***********
                              683

EZH1_HUMAN      QADALKYVGIERETDVL 747
EZH2_HUMAN      QADALKYVGIEREMEIP 746
                ************* ::
```

Supplementary Fig. 7. Sequence alignment of the SET domain of EZH1 and EZH2

Lysine residue interacting with the PDS loop upon SUZ12 phosphorylation at S583 is highlighted in a blue box.

Supplementary Fig. 8

Gene Name	Sample Name	AA Mutation	CDS Mutation	Primary Tissue	Histology	Pubmed ID	Somatic Status	Sample Type
**EZH2**	P-0007319-T01-IM5	p.K688N	c.2064A>C	Penis	Carcinoma	28481359	Confirmed Somatic	Tumour Sample
**EZH2**	EXTERN_MELA_20140530_004	p.K688E	c.2062A>G	Skin	Malignant melanoma	28467829	Confirmed Somatic	Tumour Sample

Gene Name	Sample Name	AA Mutation	CDS Mutation	Primary Tissue	Histology	Pubmed ID	Somatic Status	Sample Type
**SUZ12**	PT19_2	p.H567Y	c.1699C>T	Skin	Carcinoma	25759019	Confirmed Somatic	Tumour Sample
**SUZ12**	DLD1	p.S568G	c.1702A>G	Large intestine	Carcinoma	24755471	Previously Reported	Cultured
**SUZ12**	HCT-15	p.S568G	c.1702A>G	Large intestine	Carcinoma	23856246	Confirmed Somatic	Cultured
**SUZ12**	HCT-15	p.S568G	c.1702A>G	Large intestine	Carcinoma	24755471	Previously Reported	Cultured
**SUZ12**	HCT8	p.S568G	c.1702A>G	Large intestine	Carcinoma	24755471	Previously Reported	Cultured
**SUZ12**	P-0009618-T01-IM5	p.D582H	c.1744G>C	Urinary tract	Carcinoma	28481359	Confirmed Somatic	Tumour Sample
**SUZ12**	TCGA-XF-A9SP-01	p.S583I	c.1748G>T	Urinary tract	Carcinoma	–	Confirmed Somatic	Tumour Sample

Supplementary Fig. 8. Relevant cancer mutations

Information on cancer mutations of EZH2 and SUZ12 residues involved in the phosphoserine-centered interaction network was retrieved from COSMIC (https://cancer.sanger.ac.uk/cosmic). Amino acid number for EZH2 is based on isoform 2. K688 in EZH2 isoform 2 corresponds to K683 in isoform 1.

Supplementary Fig. 9

Gong L et al.

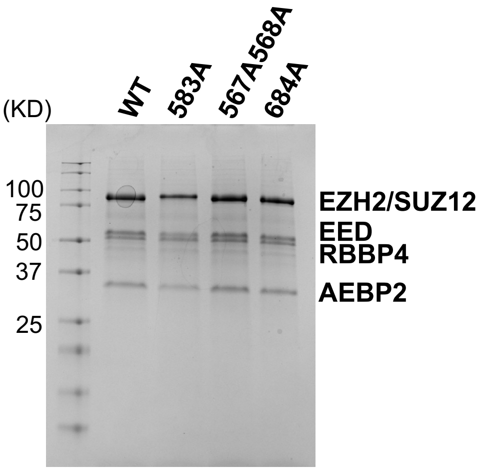

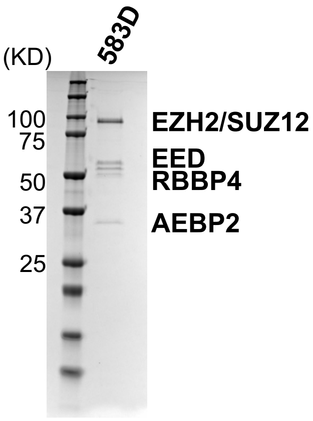

Supplementary Fig. 9. Purified PRC2-5m

PRC2-5m expressed in HEK293T cells were purified and checked on the gel stained by Coomassie blue.

Source data are provided as a Source Data file.

Supplementary Fig. 10

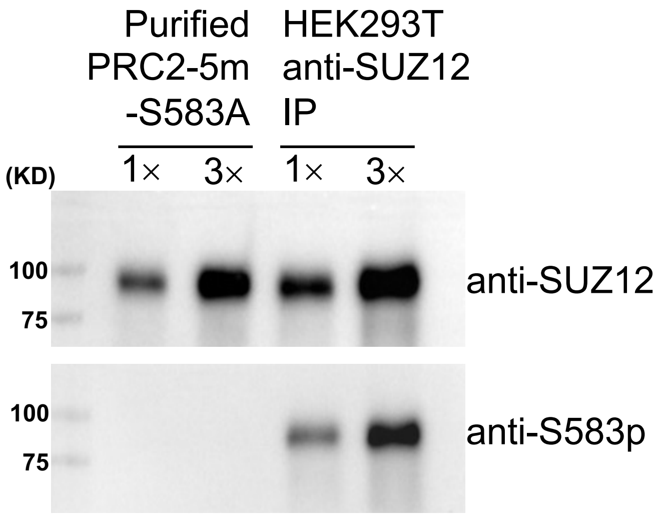

Supplementary Fig. 10. Endogenous phosphorylated SUZ12 not detected in the purified PRC2-5m

PRC2-5m-S583A was expressed in HEK293T cells. Phosphorylated SUZ12S583 was not present in the purified complex. Endogenous SUZ12 from HEK293T cell lysates served as the positive control. A representative of two independent experiments is shown.

Source data are provided as a Source Data file.

Supplementary Fig. 11

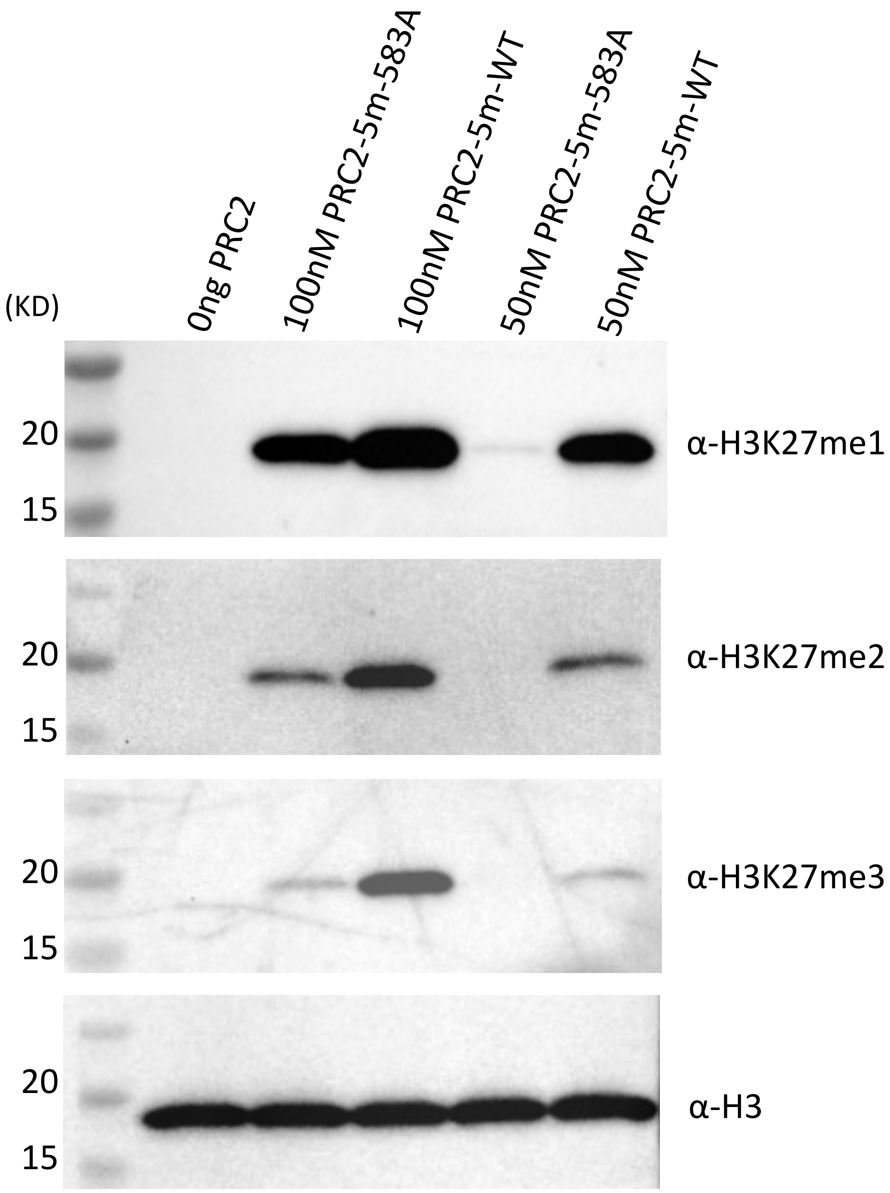

Supplementary Fig. 11. Effect of the S583A mutation of SUZ12 on all three methylation states of histone H3K27

Antibodies specific to H3K27me1, H3K27me2, or H3K27me3 were used to examine the levels of different H3K27 methylation states. Mononucleosomes were used as the substrate. Assays were performed under SAM limiting conditions. All methylation states were affected by the S583A mutation. A representative of three independent experiments is shown.

Source data are provided as a Source Data file.

Supplementary Fig. 12

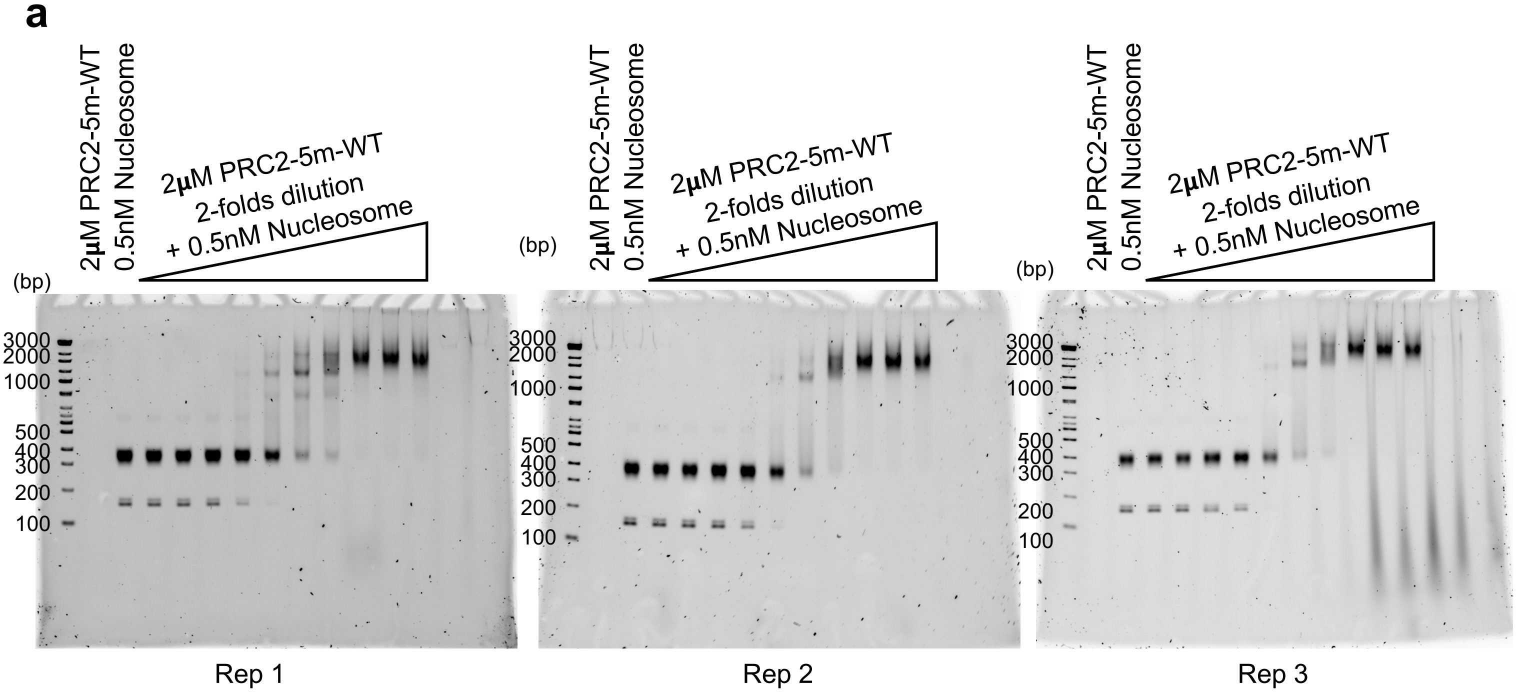

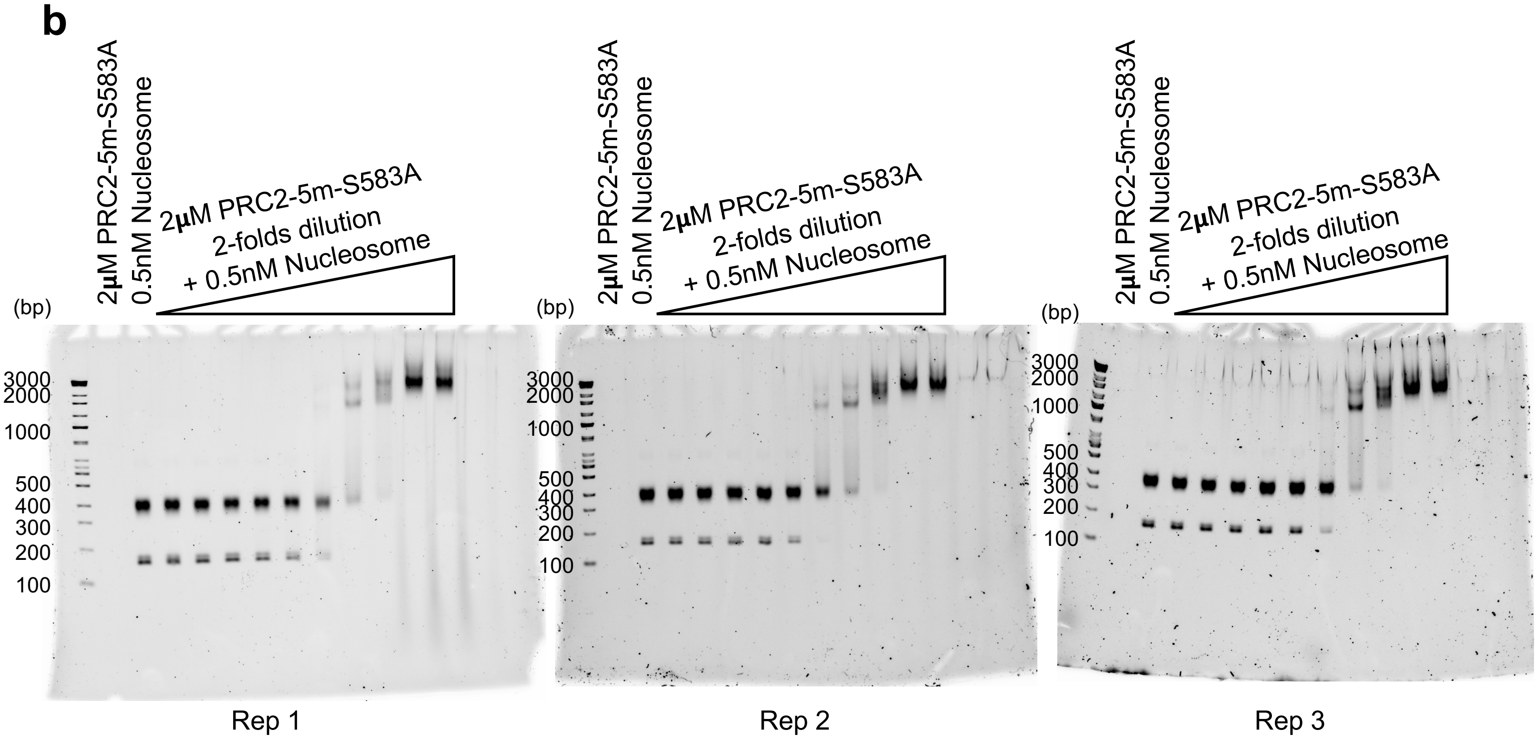

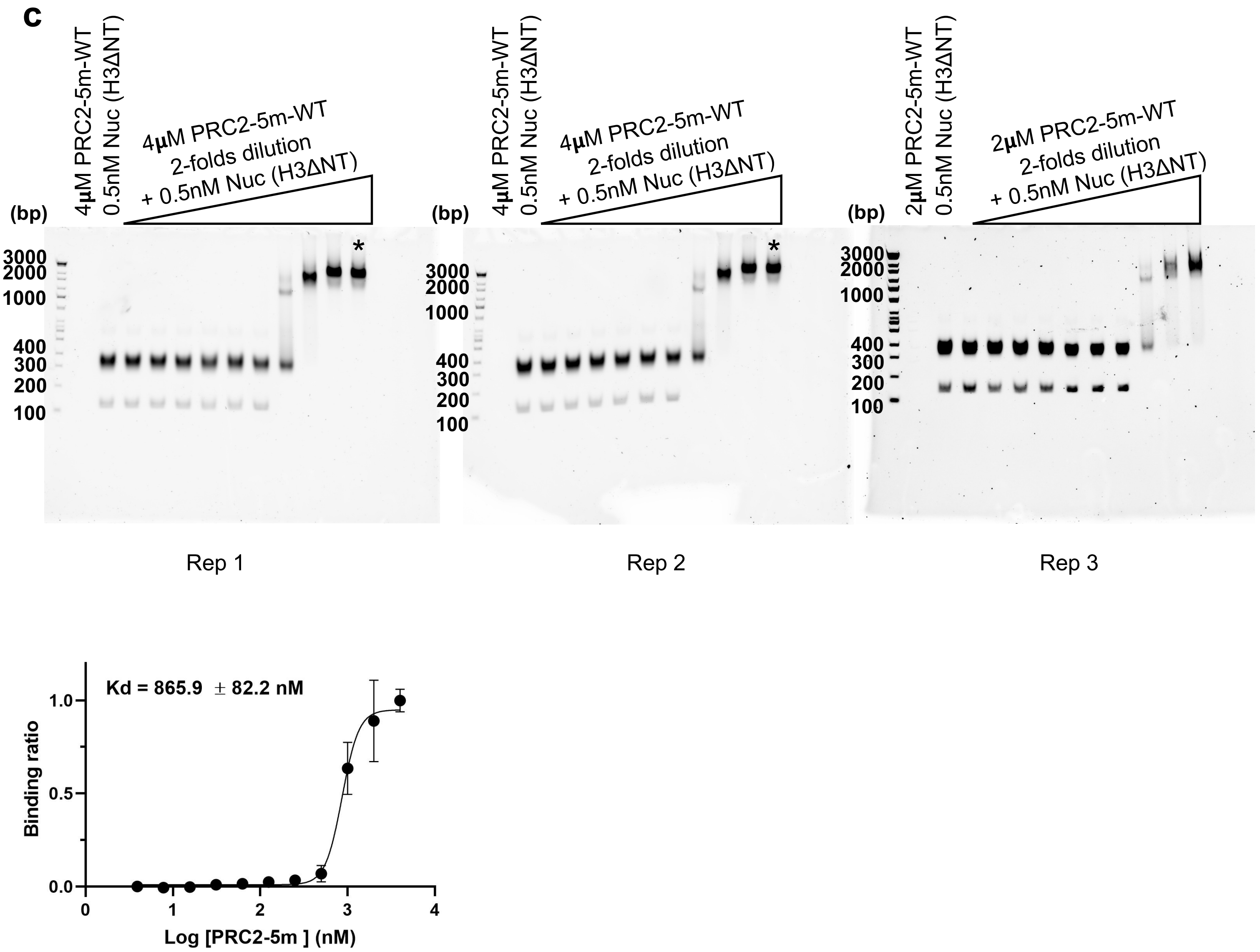

Supplementary Fig. 12. Native gel shift nucleosome binding assays

Free and PRC2-5m-bound nucleosomes were stained by SYBR Gold. Band intensities were quantified by ImageJ. In **a** and **b**, intact 147-bp ‘601’ mononucleosomes were used. Binding affinities calculated from **a** and **b** are presented in the main figure. In **c**, mononucleosomes lacking the N-terminal 1-27 residues of histone H3 were used. The binding affinity calculated from **c** is displayed below the gels (gel shift bands marked by an asterisk were not included for the calculation).

Source data are provided as a Source Data file.

Supplementary Fig. 13

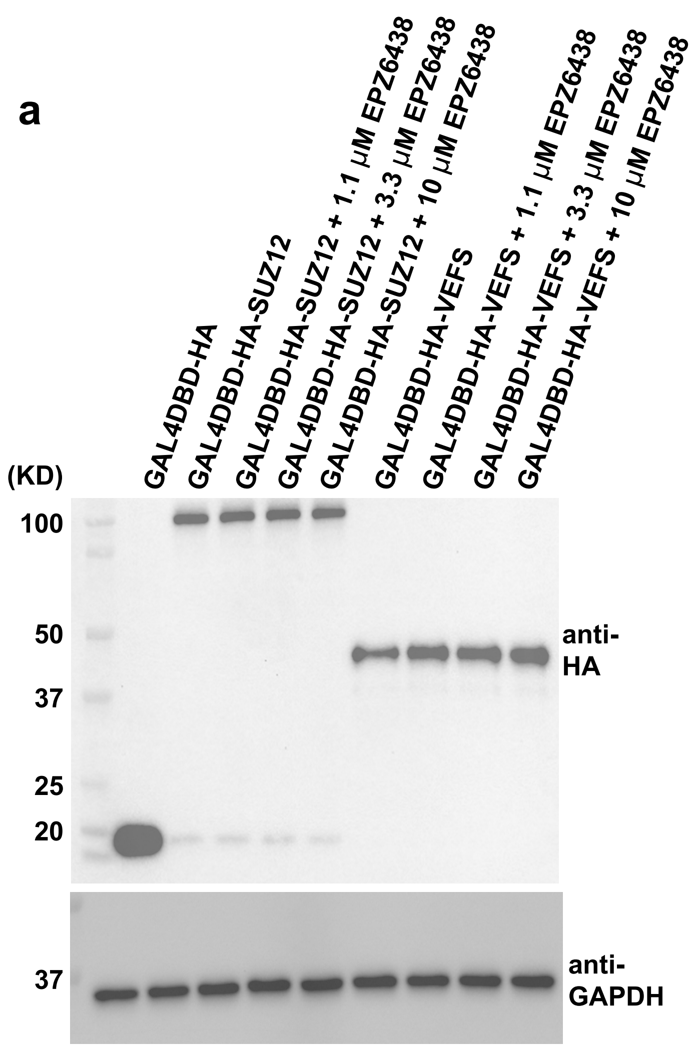

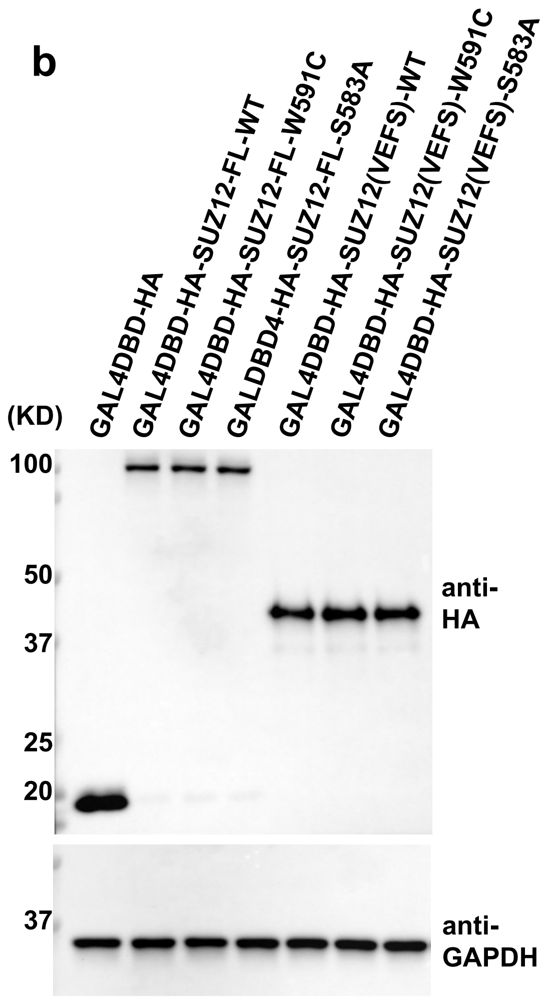

Supplementary Fig. 13. Protein expression in reporter gene repression

Transiently expressed proteins mediating reporter gene repression were examined by SDS-PAGE and Western blot. A representative of three independent experiments is shown.

Source data are provided as a Source Data file.

Supplementary Fig. 14

a

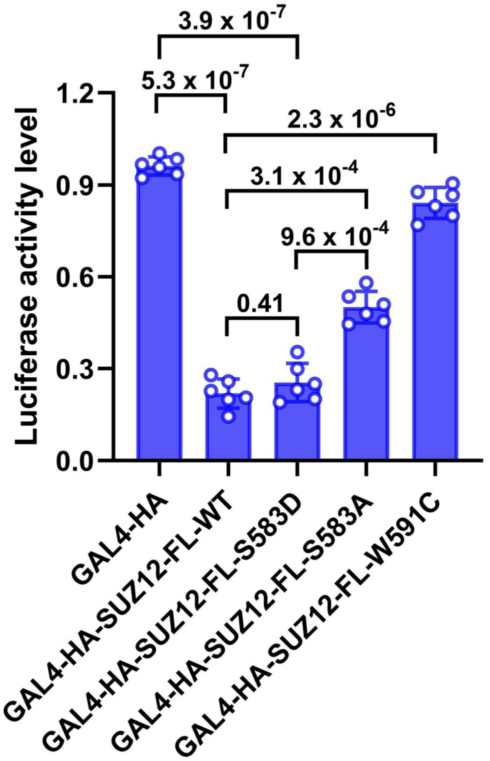

b

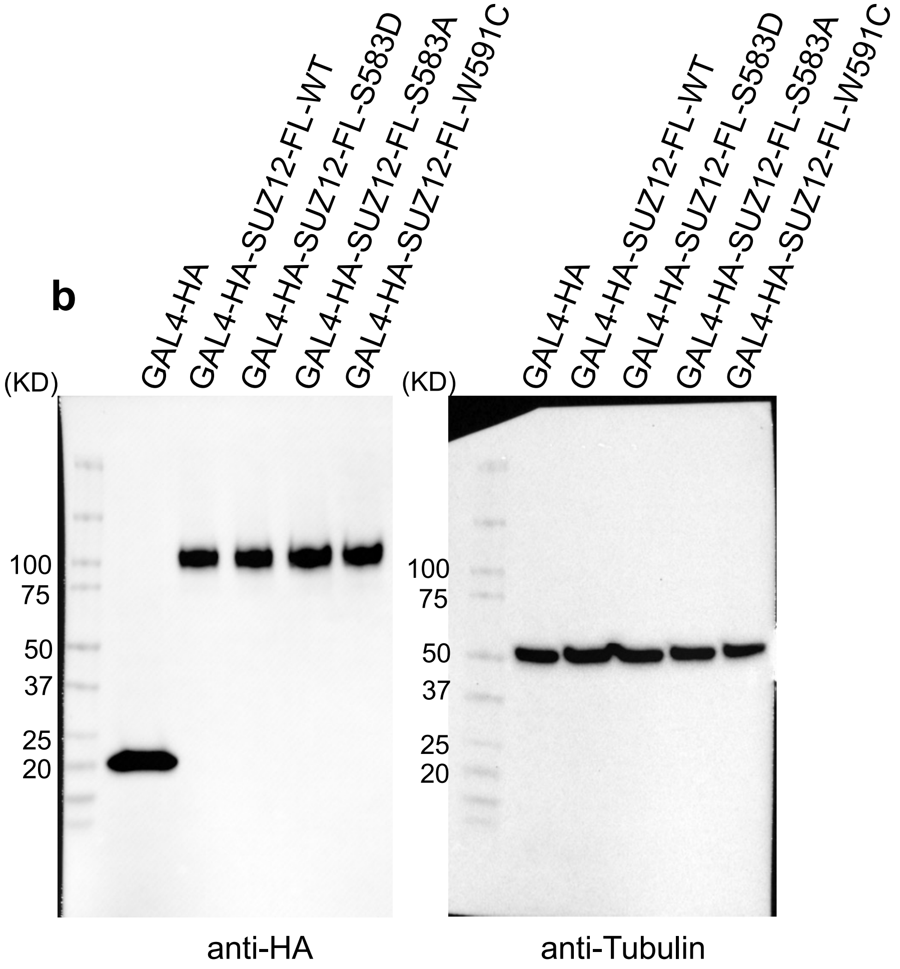

Supplementary Fig. 14. Effect of the S583D phosphomimetic mutation of SUZ12 on reporter gene repression

a. Reporter gene repression by SUZ12-FL harboring the S583D phosphomimetic mutation. Degrees of reporter gene repression were compared among the GAL4-HA (negative control), GAL4-HA-SUZ12-FL-WT, GAL4-HA-SUZ12-FL-S583D, GAL4-HA-SUZ12-FL-S583A, GAL4-HA-SUZ12-FL-W591C (positive control) constructs. Assays were performed in three different days with measurement of two replicate wells recorded each time. Signals were normalized to GAL4DBD-HA negative control. P values were derived from two-sided t tests performed in Microsoft Excel. n=6 biologically independent experiments. Error bars represent mean ± SEM.

b. Transiently expressed proteins mediating reporter gene repression were examined by SDS-PAGE and Western blot. A representative of three independent experiments is shown.

Source data are provided as a Source Data file.

Supplementary Fig. 15

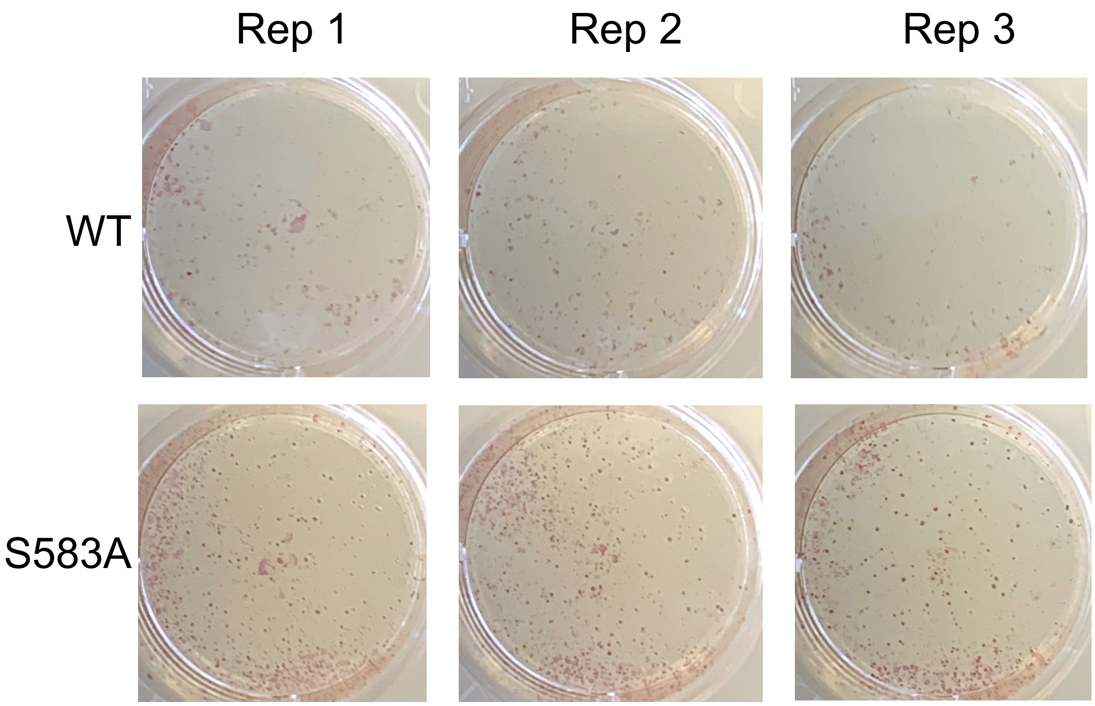

Supplementary Fig. 15. Full view of the wells in re-plating assay

Pictures were taken by a smartphone, Wells correspond to the close-up view shown in Figure 6h. Source data are provided as a Source Data file.

Supplementary Fig. 16

a (Fig. 1d)

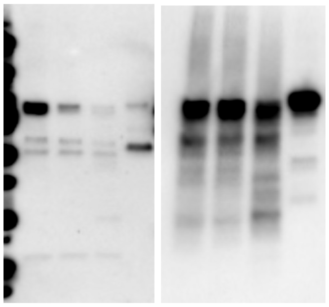

c (Fig. 2b)

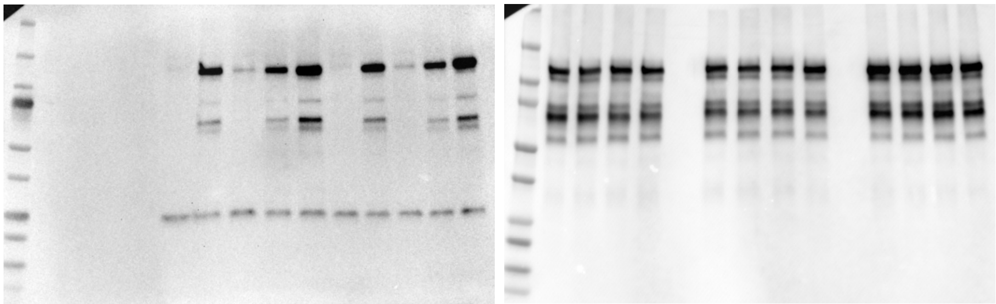

b (Fig. 1e)

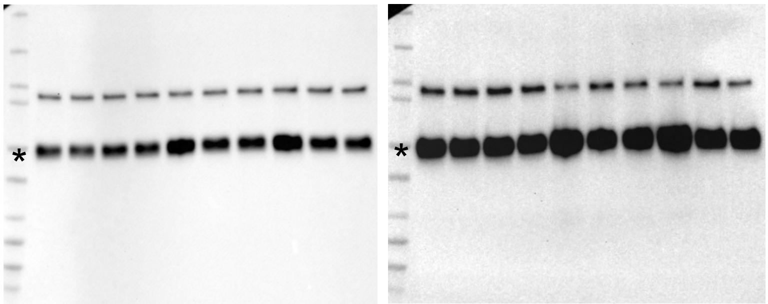

d (Fig. 2d)

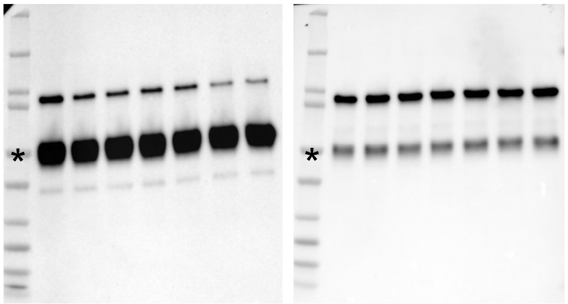

e (Fig. 4a)

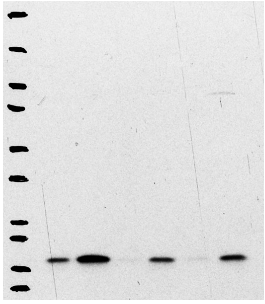

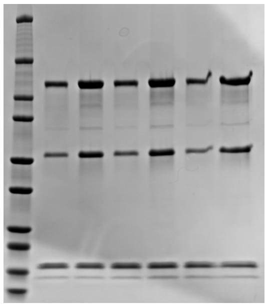

f (Fig. 2c)

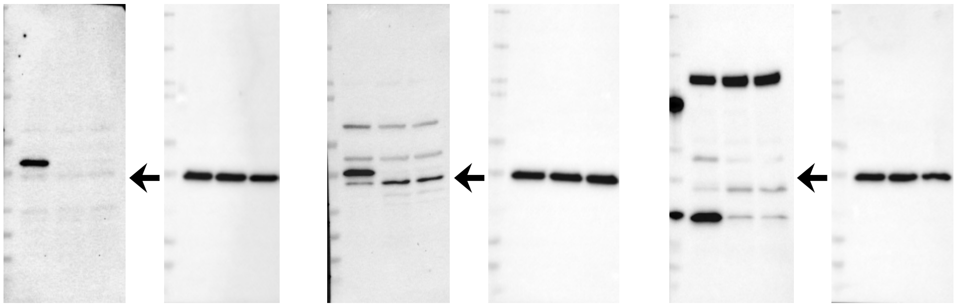

g (Fig. 4b)

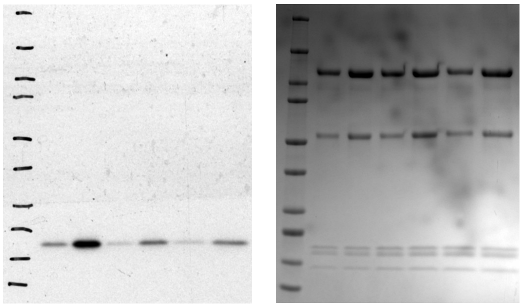

h (Fig. 4d)

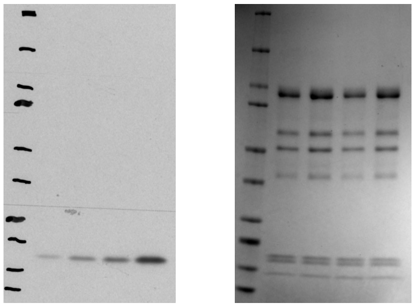

Supplementary Fig. S16. Uncropped gel images (part 1)

Uncropped gel images from the main figures are shown. Additional bands in **b** and **d** indicated by an asterisk correspond to anti-SUZ12 antibody, which was used to immunoprecipitate total SUZ12 to remove a non-specific band recognized by the anti-SUZ12SS583p antibody.

Source data are provided as a Source Data file.

Supplementary Fig. 17

a (Fig. 2e left panel)

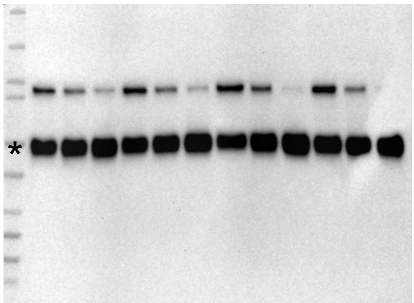

anti-S583p

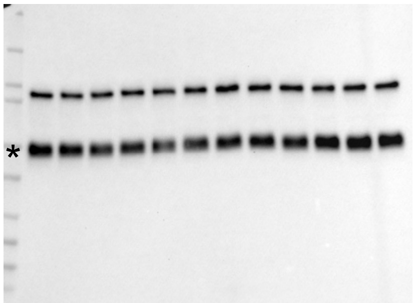

anti-SUZ12

b (Fig. 2e right panel)

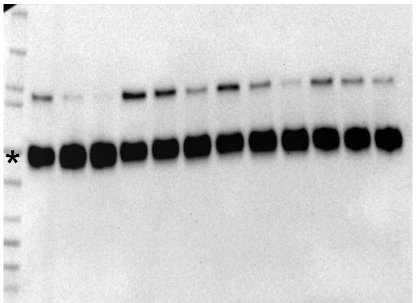

anti-S583p

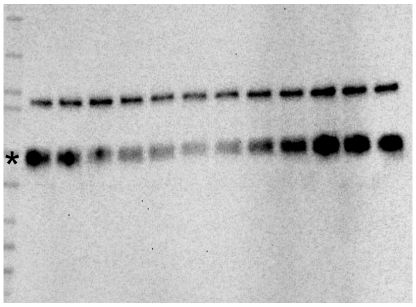

anti-SUZ12

c (Fig. 4c)

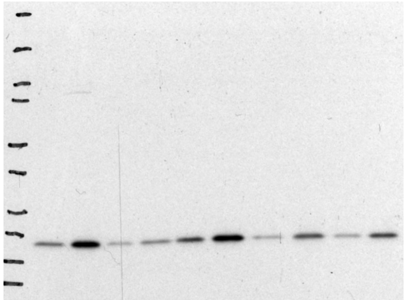

[^{3}H]

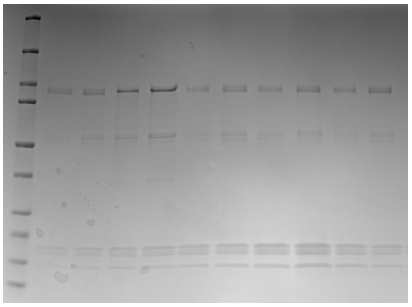

Coomassie staining

d (Fig. 4f)

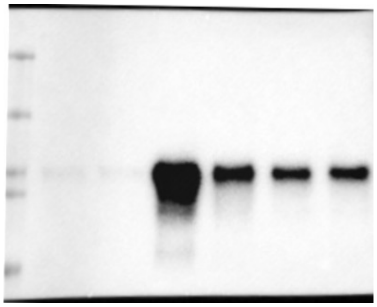

anti-EZH2

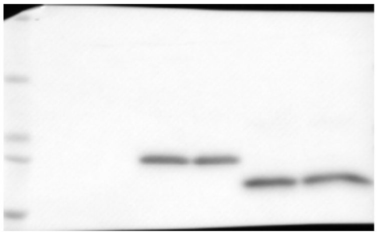

anti-H3

e (Fig. 4g)

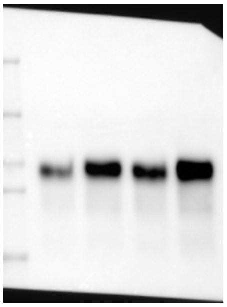

anti-EZH2

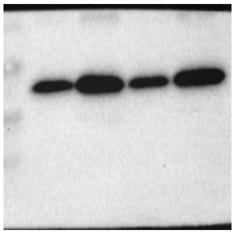

anti-
H3

Supplementary Fig. 17. Uncropped gel images (part 2)

Uncropped gel images from the main figures are shown. Additional bands in **a** and **b** indicated by an asterisk correspond to anti-SUZ12 antibody, which was used to immunoprecipitate total SUZ12 to remove a non-specific band recognized by the anti-SUZ12SS583p antibody.

Source data are provided as a Source Data file.

Supplementary Fig. 18

a (Fig. 6a)

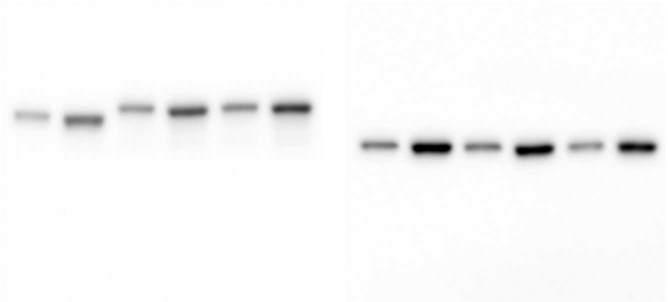

c (Fig. 6c)

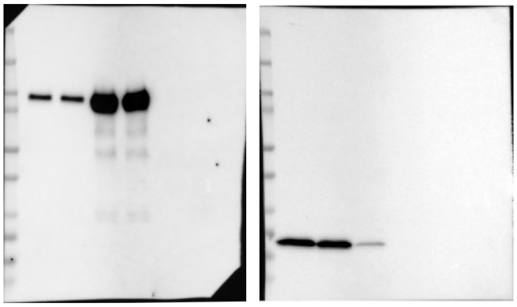

b (Fig. 6b)

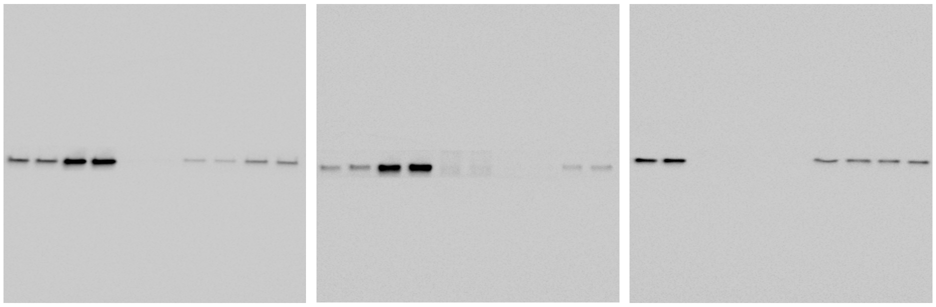

Supplementary Fig. 18. Uncropped gel images (part 3)

Uncropped gel images from the main figures are shown.

Source data are provided as a Source Data file.

Supplementary Fig. 19

a (Fig. S2)

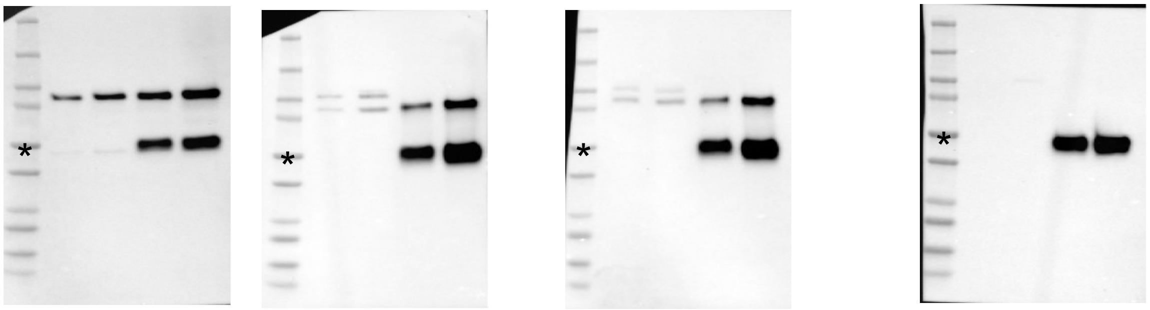

b (Fig. S3)

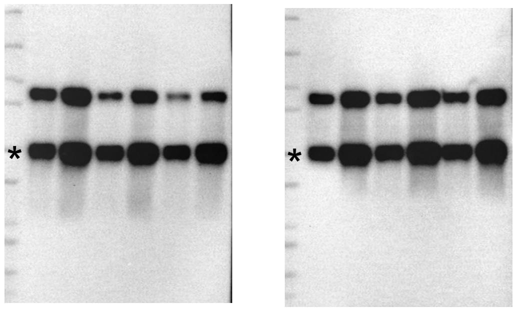

c (Fig. S6)

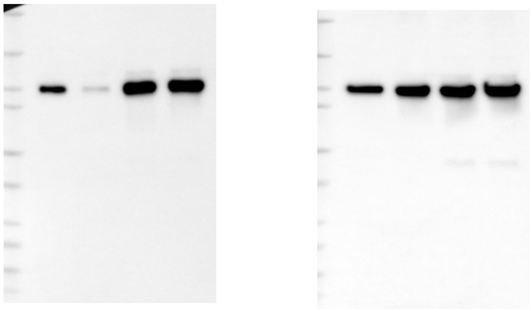

d (Fig. S11)

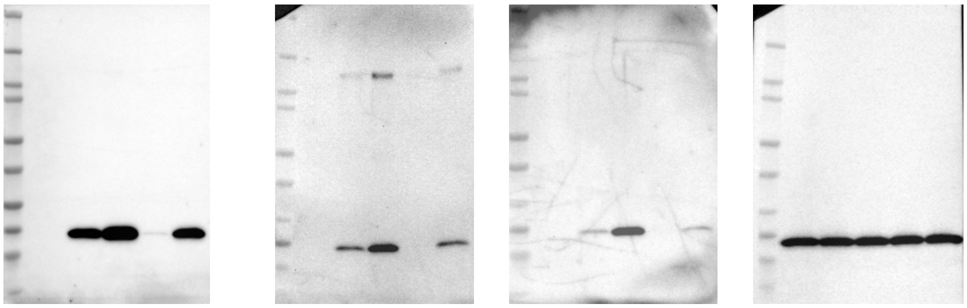

e (Fig. S13 left panel)

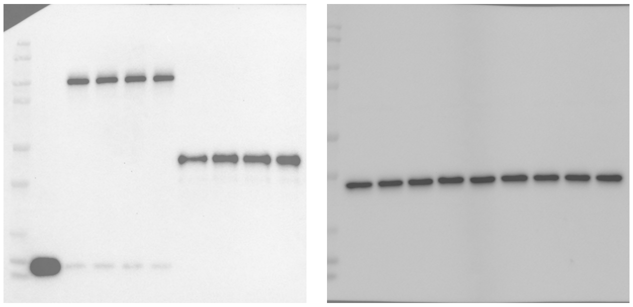

f (Fig. S13 right panel)

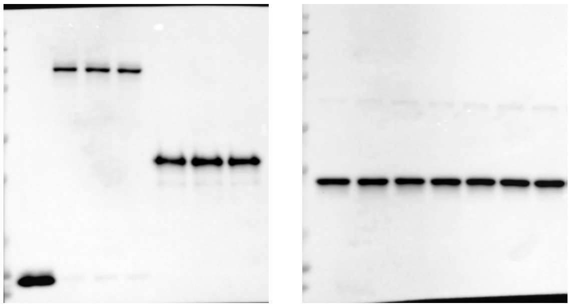

Supplementary Fig. 19. Uncropped gel images (part 4)

Uncropped gel images from the supplementary figures are shown. Additional bands in **a** and **b** indicated by an asterisk correspond to anti-SUZ12 antibody, which was used to immunoprecipitate total SUZ12 to remove a non-specific band recognized by the anti-SUZ12SS583p antibody.

Source data are provided as a Source Data file.

Supplementary Movie 1. Phosphorylation-induced conformational change of the PDS loop

Movie of the phosphorylation-induced conformational change of the PDS loop was made by morphing between PDB:5HYN and the current structure in PyMOL.

Supplementary Table 1. Diffraction Data Collection and Structure Refinement Statistics

**Data collection**	
Wavelength (Å)	0.979
Resolution range (Å)	50-3.00 (3.05-3.00)
Space group	C 1 2 1
a, b, c (Å)	231.9, 64.4, 254.5
α, β, γ (°)	90.0, 109.9, 90.0
R_{pim} (%)	4.3 (81.6)
Mean I/σ	19.2 (1.4)
$CC_{1/2}$(%)	99.5 (60.2)
Completeness (%)	99.2 (99.3)
Redundancy	6.6 (6.8)
**Refinement**	
Number of reflections	61821
R_{work}/R_{free}	0.199 /0.235
Number of non-hydrogen atoms	16191
RMS(bonds)	0.012
RMS(angles)	1.63
Ramachandran favored (%)	94.49
Ramachandran allowed (%)	4.79
Ramachandran outliers (%)	0.72

Supplementary Table 2. shRNA sequences used for CK2 knockdown.

Name	Sigma TRC number	Sequences
shCK2α #1	TRCN0000320928	CCGGATTACCTGCAGGTGGAATATTCTCG AGAATATTCCACCTGCAGGTAATTTTTTG
shCK2α #2	TRCN0000350294	CCGGTGGAATATTTCATGGACAAATCTCG AGATTTGTCCATGAAATATTCCATTTTTG
shCK2α' #1	TRCN0000318690	CCGGCCTCACAATGTCATGATAGATCTCG AGATCTATCATGACATTGTGAGGTTTTTG
shCK2α' #2	TRCN0000000614	CCGGCTGGGACAACATTCACGGAAACTCG AGTTTCCGTGAATGTTGTCCCAGTTTTT
shCK2β #1	TRCN0000231582	CCGGCAGGCAGCCGAGATGCTTTATCTCG AGATAAAGCATCTCGGCTGCCTGTTTTTG
shCK2β #2	TRCN0000003796	CCGGTGGTTTCCCTCACATGCTCTTCTCG AGAAGAGCATGTGAGGGAAACCATTTTT

Supplementary Table 3. List of primers used for ChIP-qPCR

Locus	Forward primer sequence (5' to 3')	Reverse primer sequence (5' to 3')
HOXA7	GAGAGGTGGGCAAAGAGTGG	CCGACAACCTCATACCTATTCCTG
HOXD12	GATGTGTGAGCGCAGTCTCT	GCTGCGAGGGTATGAGATGG
GATA4	CTCTCCCGAGCTCACTTCAAGG	GGAGAAGGTGACCTCGCACAC
FGF5	AGGGACGGTCAAGATTCCTT	AGAACCAGCAGAGTCCCAGA
NES	CTGGAGCGCGAGTTAGAGG	TGGCCAGCTCCTCGACTT
NANOG	GGCATGGTGGTAGACAAGCC	TTAGTAAGTTGGTCCATGCTTTGG
