# Supplementary material for: CK2-mediated phosphorylation of SUZ12 promotes PRC2 function by stabilizing enzyme active site
Source: Nat Commun. 2022 Nov 9;13:6781. doi: 10.1038/s41467-022-34431-1 (PMC9645763; doi:10.1038/s41467-022-34431-1)
Supplement: Supplementary file 4 — Reporting Summary [file 41467_2022_34431_MOESM4_ESM.pdf]

nature portfolio

Corresponding author(s): Xin Liu

Last updated by author(s): Oct 9, 2022

# Reporting Summary

Nature Portfolio wishes to improve the reproducibility of the work that we publish. This form provides structure for consistency and transparency in reporting. For further information on Nature Portfolio policies, see our Editorial Policies and the Editorial Policy Checklist.

## Statistics

For all statistical analyses, confirm that the following items are present in the figure legend, table legend, main text, or Methods section.

| n/a | Confirmed | |
|---|---|---|
| ☐ | ☒ | The exact sample size (*n*) for each experimental group/condition, given as a discrete number and unit of measurement |
| ☐ | ☒ | A statement on whether measurements were taken from distinct samples or whether the same sample was measured repeatedly |
| ☐ | ☒ | The statistical test(s) used AND whether they are one- or two-sided<br>*Only common tests should be described solely by name; describe more complex techniques in the Methods section.* |
| ☒ | ☐ | A description of all covariates tested |
| ☒ | ☐ | A description of any assumptions or corrections, such as tests of normality and adjustment for multiple comparisons |
| ☐ | ☒ | A full description of the statistical parameters including central tendency (e.g. means) or other basic estimates (e.g. regression coefficient) AND variation (e.g. standard deviation) or associated estimates of uncertainty (e.g. confidence intervals) |
| ☐ | ☒ | For null hypothesis testing, the test statistic (e.g. *F*, *t*, *r*) with confidence intervals, effect sizes, degrees of freedom and *P* value noted<br>*Give P values as exact values whenever suitable.* |
| ☒ | ☐ | For Bayesian analysis, information on the choice of priors and Markov chain Monte Carlo settings |
| ☒ | ☐ | For hierarchical and complex designs, identification of the appropriate level for tests and full reporting of outcomes |
| ☒ | ☐ | Estimates of effect sizes (e.g. Cohen's *d*, Pearson's *r*), indicating how they were calculated |

*Our web collection on statistics for biologists contains articles on many of the points above.*

## Software and code

Policy information about availability of computer code

| | |
|---|---|
| Data collection | Crystal diffraction data were collected at APS beamline 19-ID. MS scans were acquired in Orbitrap and spectra were obtained in the ion trap for each full spectrum acquired. |
| Data analysis | Diffraction data were processed with HKL2000. Molecular replacement was performed using CCP4 suit. The structure was refined by REFMAC5 and autoBUSTER 2.10.4 and refinement statistics was generated by PHENIX 1.14. Model building and iterative refinement were carried out by using Coot 0.9.6. Structure figures were generated by PyMOL 2.5.2. Raw MS data files were analyzed using Proteome Discoverer v2.4 SP1. Statistical analysis was performed in Microsoft Excel and GraphPad Prism v9.2.0. Images were quantified in ImageJ 1.53a. |

For manuscripts utilizing custom algorithms or software that are central to the research but not yet described in published literature, software must be made available to editors and reviewers. We strongly encourage code deposition in a community repository (e.g. GitHub). See the Nature Portfolio guidelines for submitting code & software for further information.

## Data

Policy information about availability of data

All manuscripts must include a data availability statement. This statement should provide the following information, where applicable:

- Accession codes, unique identifiers, or web links for publicly available datasets
- A description of any restrictions on data availability
- For clinical datasets or third party data, please ensure that the statement adheres to our policy

The crystal structures described in this study has been deposited in the Protein Data Bank under the accession number 7TD5. The LC-MS/MS data files have been deposited to the ProteomeXchange Consortium (http://proteomecentral.proteomexchange.org) via the MassIVE partner repository with the dataset identifier MSV000088683. Previously published crystal structures 5HYN and 5IJ7 are available at Protein Data Bank https://www.rcsb.org/

# Field-specific reporting

Please select the one below that is the best fit for your research. If you are not sure, read the appropriate sections before making your selection.

☒ Life sciences ☐ Behavioural & social sciences ☐ Ecological, evolutionary & environmental sciences

For a reference copy of the document with all sections, see nature.com/documents/nr-reporting-summary-flat.pdf

# Life sciences study design

All studies must disclose on these points even when the disclosure is negative.

| | |
|---|---|
| Sample size | Sample sizes used in this study were determined according to our experience and published literatures and are stated in the manuscript. For experiments where an error bar is calculated, a sample size of at least 3 was used. |
| Data exclusions | No data were excluded from analysis. |
| Replication | ChIP q-PCR was performed with two biological replicates each with three technical replicates. Reporter assay was repeated six times with two biological replicates. Enzyme activity assay were repeated for three times. mESC replating assay was repeated three times with different batches of mESCs. All attempts at replication were successful. All other experiments were repeated two to three times and all attempts at replication were successful. |
| Randomization | Randomization is not applicable. Only one person was involved in each experiment. |
| Blinding | Blinding is not applicable. Only one person was involved in each experiment. |

# Reporting for specific materials, systems and methods

We require information from authors about some types of materials, experimental systems and methods used in many studies. Here, indicate whether each material, system or method listed is relevant to your study. If you are not sure if a list item applies to your research, read the appropriate section before selecting a response.

## Materials & experimental systems

| n/a | Involved in the study |
|---|---|
| ☐ | ☒ Antibodies |
| ☐ | ☒ Eukaryotic cell lines |
| ☒ | ☐ Palaeontology and archaeology |
| ☒ | ☐ Animals and other organisms |
| ☒ | ☐ Human research participants |
| ☒ | ☐ Clinical data |
| ☒ | ☐ Dual use research of concern |

## Methods

| n/a | Involved in the study |
|---|---|
| ☒ | ☐ ChIP-seq |
| ☒ | ☐ Flow cytometry |
| ☒ | ☐ MRI-based neuroimaging |

## Antibodies

Antibodies used

Rabbit anti-SUZ12, Cell Signaling, WB: 1:1000, CST3737
Rabbit anti-CK2a, GeneTex, WB: 1:500, GTX107897
Rabbit anti-CK2a', Bethyl, WB: 1:500, A300-199A
Rabbit anti-CK2b, Bethyl, WB: 1:500, A301-984A
Rabbit anti-phosphoerine, Abcam, WB: 1:500, Ab9332
Mouse anti-GAPDH, Invitrogen, WB: 1:1000, MA515738
Rabbit anti-EZH2, Cell Signaling, WB: 1:1000, CST5246
Rabbit anti-H3, Cell Signaling, WB: 1:5000, CST4499
Rabbit anti-HA tag, Cell Signaling, WB: 1:1000, CST3724
Mouse anti-FLAG tag, Sigma, WB: 1:1000, F1804
Mouse anti-FLAG tag, Sigma, ChIP: 1:500, F1804
Rabbit anti-H3K27me3, Cell signaling, WB: 1:1000, CST9733
Rabbit anti-H3K27me3, Cell signaling, ChIP: 1:200, CST9733
Rabbit anti-b-Tubulin, Cell Signaling, WB: 1:1000, CST2128
Rabbit anti-H3K27me2, Millipore, WB: 1:500, 07-452
Rabbit anti-H3K27me1, Millipore, WB: 1:500, 07-448
Rabbit antibody specific for SUZ12S583p, WB: 1:1000, This paper

Validation

The commercial antibodies were validated based on the information from manufactures' instructions. The antibody against SUZ12S583p made in this study was validated by performing western blot and dot blot and was not validated for the species. We validated the antibody for SUZ12 (Cell Signaling, Cat No. 3737) using knockout. This antibody was also validated by the manufacturer

using SimpleChIP® Enzymatic Chromatin IP Kits. We validated antibodies for CK2a (GeneTex, Cat No.GTX107897), CK2a' (Bethyl, Cat No. A300-199A), and anti-CK2b (Bethyl, Cat No. A301-984A) using knockdown. The antibody for EZH2 (Cell Signaling, Cat No. 5246), HA tag (Cell Signaling, Cat No. 3724), H3K27me3 (Cell signaling, Cat No. 9733), and b-Tubulin (Cell Signaling, Cat No. 2128) have been validated using SimpleChIP® Enzymatic Chromatin IP Kits by the manufacture. The antibody for FLAG tag (Sigma,Cat No. F1804) was validated by previous publications: PMID 32312989 and PMID 33247121. The antibody for H3 (CST, 4499S) has been validated for western blot by the manufacturer and > 400 previous publications: https://www.cellsignal.com/products/primary-antibodies/histone-h3-d1h2-xp-rabbit-mab/4499. The antibody for GPADH (Invitrogen, Cat No. MA515738) has been validated for western blot by the manufacturer and > 200 previous publications: https://www.thermofisher.com/antibody/product/MA5-15738.html?CID=AFLCA-MA5-15738. The antibody for phosphoserine (Abcam, Cat. No.ab9332) has been validated by the manufacturer and > 200 previous publications: https://www.abcam.com/phosphoserine-antibody-ab9332.html?productWallTab=ShowAll.

## Eukaryotic cell lines

Policy information about cell lines

| | |
|---|---|
| Cell line source(s) | HEK293T, A172, MDA-MB-231, U118MG, LNCap, 22RV1, NT2/D1, MCF-7, MCF10A, and BT-474 were from ATCC. SUZ12 knockout mESC line was a kind gift from Dr. Kristian Helin (Institute of Cancer Research) and is not commercially available. This cell line has been described previously in Højfeldt et al. Nat Struct Mol Biol . 2018. SUZ12-WT and SUZ12-S583A were delivered to Suz12 KO cells with lentiviruses. |
| Authentication | mESC KO cell line was validated by Sanger sequencing of genomic DNA and western blotting. SUZ12-WT and SUZ12-S583A cell lines were assessed by morphology, western blotting, ES marker gene expression and alkaline phosphatase staining. HEK293T, A172, MDA-MB-231, and NT2/D1 were authenticated. U118MG, LNCap, 22RV1, NT2/D1, MCF-7, MCF10A, and BT-474 were not authenticated. |
| Mycoplasma contamination | All cell lines tested were negative for mycoplasma. Cells were tested every three months. |
| Commonly misidentified lines (See ICLAC register) | No commonly misidentified cell lines were used. |
